# Midwives’ perspective on participation of pregnant individuals planning an elective caesarean section delivery in antenatal classes in Germany: a qualitative interview study

**DOI:** 10.1186/s12884-026-09676-z

**Published:** 2026-07-21

**Authors:** Sandra Jaax, Jessica Breuing, Barbara Prediger, Nadja Könsgen

**Affiliations:** Institute for Research in Operative Medicine, Faculty of Health, School of Medicine, University of Witten/Herdecke, Ostmerheimer Str. 200, Building 38, Cologne, 51109 Germany

**Keywords:** Prenatal classes, Childbirth Education Classes, Childbirth Preparation Classes, Cesarean Sections, Pregnancy, Germany, Qualitative Study

## Abstract

**Background:**

Studies indicate that pregnant individuals sometimes feel inadequately prepared for caesarean section (CS) in antenatal classes. This suggests that the antenatal classes may not sufficiently address the information needs of those planning an elective CS. As antenatal classes provide valuable information beyond preparation for vaginal birth, they may nevertheless be relevant for this group. This study aims to examine how antenatal classes in Germany address elective CS and what factors influence the participation of pregnant individuals planning to have an elective CS according to midwives.

**Methods:**

We conducted a qualitative study using semi-structured interviews with midwives, who were sampled via a convenience sampling technique. The eligibility criteria required midwives currently offering antenatal classes who had conducted at least one antenatal class in Germany that included a pregnant individual planning an elective CS. Recruitment was carried out via telephone or email and through midwifery associations. The 15 interviews took place between October and December 2023. We performed data analysis using a content-structured approach (Mayring), employing MAXQDA 2022 (VERBI Software, Berlin, Germany).

**Results:**

Midwives reported, as a barrier to participation, that pregnant individuals preparing for an elective CS often assume that antenatal classes are not relevant for them. This is partly due to a lack of information regarding the importance of their participation as well as insufficient awareness about the content of antenatal classes. Moreover, midwives noted that, as another barrier of participation, other participants and midwives in antenatal classes sometimes respond negatively to the decision of having a CS. All midwives generally take CS as a topic into account and most generally discuss the content of elective CS. The majority of midwives in the study considered addressing the informational needs of pregnant individuals preparing for an elective CS to be important. The idea of offering a separate course was subject to extensive discussion during the interviews. Some participants raised concerns regarding its practical feasibility.

**Conclusions:**

A more comprehensive education of all pregnant individuals regarding the content of antenatal classes is necessary. Tailored course formats could improve access to and relevance of antenatal classes for pregnant individuals planning an elective CS.

**Supplementary Information:**

The online version contains supplementary material available at 10.1186/s12884-026-09676-z.

## Background

Antenatal classes are defined as structured programs for expectant parents that provide information and skills to prepare for childbirth, parenthood and postnatal care [1]. Pregnant individuals attend antenatal classes to reduce their fear of childbirth and labour [1], to prepare them for the process of giving birth and becoming parents, and to feel confident in caring for a newborn [2]. Additionally, antenatal classes provide a social network and an opportunity to share experiences [3].

In Germany, antenatal classes are mainly provided by midwives. In addition to topics and practical exercises related to vaginal birth, antenatal classes cover other topics related to pregnancy and preparation for parenthood. These include information about the postnatal period, breastfeeding and newborn care.

Out of a total of 667,705 individuals who gave birth in Germany in 2023, 217,852 had a caesarean section (CS). This represents a CS rate of 32.6% [4].

A mixed-methods study from Switzerland found that, of the mothers who rated antenatal classes as moderately to not helpful at all, 19% had a CS and received no information on the procedure [5]. This raises the question of how antenatal classes can be designed to appeal to women planning a CS and meet their information needs. As antenatal classes cover topics that go beyond vaginal birth, they are also relevant to this group of pregnant individuals. Furthermore, it is important to include CS in antenatal classes for pregnant individuals planning a vaginal birth, because they may unexpectedly find themselves in a situation where they require an unplanned CS and benefit from proper preparation for a CS. Against this background, it is also important to determine under what circumstances pregnant individuals planning an elective CS attend antenatal classes.

Our aim was to examine how antenatal classes address elective CS and what influences the participation of pregnant individuals planning an elective CS.

## Methods

### Ethical considerations

The Witten/Herdecke University Ethical Committee (S-240/2023) approved this qualitative study.

### Design

This descriptive cross-sectional study was conducted to explore midwives’ perspectives on how antenatal classes address elective CS and what influences the participation of pregnant individuals planning an elective CS. The current study builds on a study protocol that was registered and published on protocols.io [6] prior to data collection. Accordingly, we adapted sections of the introduction and methods with transparent text recycling following the Text Recycling Research Project as instrument of guidance [7]. Moreover, we used the Consolidated Criteria for Reporting Qualitative Research (COREQ) [8] to report the present study (Supplement 2: COREQ checklist).

### Study setting

The study was conducted in Germany. Participants were midwives practicing in Germany, operating within the German maternity care system.

### Sample and sampling

We included a convenience sample of midwives who have experience in conducting antenatal classes in Germany and who offered antenatal classes at the time of recruitment. Only midwives who had experience with pregnant individuals preparing for an elective CS attending at least one of their antenatal classes were included. It was possible to recruit enough midwives who met these criteria. Elective CS included both the maternal request CS and the elective CS due to a medical indication. If it had not been possible to recruit enough midwives with experience, we would have recruited midwives with no experience of pregnant individuals preparing for an elective CS in their antenatal classes. Sample size was determined by data sufficiency, with recruitment continuing until data were deemed adequate to comprehensively address the research questions. We used convenience sampling and data sufficiency because the study was part of a Master’s thesis, which meant that we had limited time and resources.

To recruit midwives, midwives practicing in Germany were contacted by phone or email, or via invitation from midwifery associations. Recruitment took place from October to December 2023. We obtained the contact details of the midwives from the midwives’ list of the GKV-Spitzenverband (National Association of Statutory Health Insurance Funds) [9]. Influencers in the field of midwifery science were contacted via Instagram and invited to share information about the study. This included both midwives who are active on social media and organizations. In addition, we leveraged the Institute for Research in Operative Medicine (IFOM) network to support recruitment and disseminated information about the study via online messaging platforms and social media accounts. There were no financial incentives for participation.

Midwives who were interested in participating received an email with the following study documents, prior to the interview: participation information, consent form and privacy statement. Furthermore, the email requested information on the characteristics relevant to heterogeneous composition of the sample, such as age, gender, and professional experience in conducting antenatal classes.

A total of 31 midwives contacted us, comprising 18 reached by phone (31% of midwives contacted via phone were interested), eight who responded via email (4% of midwives contacted via mail were interested), and five identified through other recruitment channels (it is not possible to calculate percentage of interested midwives). Seven of the 31 interested midwives could not be included because they lacked experience in providing antenatal classes or working with pregnant individuals preparing for an elective CS in their antenatal classes (*n* = 2), lacked time capacities (*n* = 4), or were uncomfortable with the way the study was conducted (participant did not want any interview/audio recordings; *n* = 1). Nine of the interested midwives did not further respond. However, data sufficiency occurred after 15 interviews had been conducted. Subsequently, we conducted no further interviews. The interviews had a mean length of 27 min (range 14–52).

### Development of the interview guide

An interdisciplinary team comprising individuals with experience in developing interview guides (JB, NK) and those without such experience (SJ) developed the semi-structured interview guide. The team members have a background in (1) health economics, (2) nutritional science, or (3) prevention, sports therapy and health management. The interview guide consisted of 6 sections: (1) General information about the antenatal classes, (2) the topic of CS in general and elective CS in the antenatal classes, (3) rationale for antenatal class attendance for those planning elective CS, (4) information needs of pregnant individuals preparing for an elective CS in antenatal classes, (5) addressing the information needs in antenatal classes, (6) factors influencing the participation of pregnant individuals preparing for elective CS in antenatal classes. The interviews were conducted in German, the translated interview guide can be found in supplementary material 1. Before starting the study, we performed a pretest of the interview guide with one midwife to ensure good comprehensibility and completeness of the questions [10]. This pretest allowed us to verify the comprehensibility of the questions, the logical flow, and the coverage of relevant topics. We subsequently adapted the wording and added explanations to make sure to the interview participants, that they should refer their answers to both the maternal request CS and the elective CS due to a medical indication. In addition, we changed the positioning of one question and added one question.

### Data collection

We conducted the pretest and the interviews by phone with the interviewer in a private environment at home or work, there was no guarantee that participants were necessarily alone at the time of the interview.

SJ, who was a Master’s student in Prevention, Sports Therapy and Health Management and an employee at IFOM at the time the interviews were carried out, conducted the semi-structured interviews as part of her Master’s thesis. At the time of the interviews, she had no prior formal training in conducting qualitative studies and was supervised by an individual with experience in conducting qualitative studies (NK). There was no relationship between the participants and the interviewer. The participants only knew that she was a Master’s student and employee at IFOM and was conducting the study as part of her master’s thesis.

We used an audio recording device to record the telephone interviews, which were then transcribed by an external service provider called Abtipper.de. Reflexivity was addressed through repeated transcript review, during which the interviewer ensured accuracy of the transcripts, familiarized with the data, and critically examined potential biases and assumptions that may have influenced data collection and interpretation. In addition, parts of the analysis were conducted by two people, thereby minimizing personal biases and assumptions. Systematic documentation of reflexive considerations in the form of field notes or a reflexivity journal was not conducted. The participants were not given the opportunity to correct or comment on the transcripts, and we did not repeat any interviews.

There were no physical or mental risks to consider during the study. Participants were informed of the content and the objectives of the study in advance. They provided written consent in advance for the conduct of interviews as well as data recording and processing.

### Data analysis

We based the interview analysis on Mayring’s qualitative content analysis [10] using MAXQDA 2022 (VERBI Software, Berlin, Germany) [11]. The aim is to structure the transcribed interviews into main and sub-categories in order to analyze the material in a controlled and step-by-step manner. The development of the main and sub-categories is based on both a deductive and an inductive approach. In the deductive approach, main categories are defined based on the interview guide. In the inductive approach, additional categories are derived from the data material. SJ and NK independently coded three interviews using the main categories. They discussed deviations to reach consensus. The interviewer then continued the first step (main categories) of coding independently. Both independently conducted the subsequent inductive coding based on 3 interviews. Following this, SJ and NK discussed the results to reach a consensus. During this step, the deductive category system from the first step was inductively refined and supplemented by further categories and subcategories. Then, the interviewer conducted the more in-depth coding independently. The coding system is provided in Supplementary Material 3.

## Results

### Participants’ characteristics

All participants were female and the mean age was 39 years (range 23–60). They had an average work experience of 11 years (range 1–25).

### Barriers and facilitators for participation in antenatal classes

Analysis of the interviews revealed several barriers and facilitators influencing the attendance of pregnant individuals preparing for an elective CS in antenatal classes. The identified themes encompassed information provided about antenatal classes, the way the topic of CS is addressed in these classes, the range of courses on offer, the choice of topics, as well as individual and social factors. Some midwives mentioned the pregnant individual’s primary maternity care provider as a barrier or facilitator to attending antenatal classes. For instance, some perceived early contact with a midwife as beneficial, while some perceived purely doctor-based care as a hindrance to pregnant individuals accessing antenatal classes. The following quote explains this in more detail:

“*Pregnant women who seek midwife care relatively early on*,* i.e. through check-ups or help with complaints*,* counselling*,* are usually very open and interested in birth preparation. I would say that pregnant women who are purely under the care of a doctor are less likely to take part in an antenatal class if they are planning a caesarean section*,* they are more likely to look for the information themselves and of course get information from their doctor. But of course the community also says: “Well*,* you’re having a caesarean section. You don’t need to do a preparation course.” And if*,* of course*,* the woman has already been in contact with the midwife by then*,* then of course you can discuss it and say: “Listen*,* you’ll find information on these and those topics”.*” Participant 8.

This suggests that models of care centred around physicians do not place a high value on attending antenatal classes for pregnant individuals planning an elective CS. This could encourage pregnant individuals who are unsure whether an antenatal class is right for them not to take part. Moreover, midwives noted that, as another barrier to participation, other participants and midwives in antenatal classes sometimes respond negatively to the decision of having a CS. For example, midwives reported that CS is sometimes perceived as inferior to vaginal birth. Further, they reported occasional attempts to persuade individuals to opt for vaginal birth. Accordingly, it was considered a facilitator if the midwife is known in advance and it is clear that the mode of birth will not be judged:

“*I think it also depends on which midwife does it. Because I sometimes get the feeling that women who have a planned caesarean section have to justify it. To other pregnant women or something. And I think that if they know the midwife*,* at least that’s my feeling*,* then they know that they won’t be judged. Can you say that? And that*,* at least*,* the midwife is very relaxed about the subject. Or knows why. Exactly*,* that it will be received differently in the course than if you go somewhere where you don’t know anyone and then you say that.*” Participant 11.

Fear of prejudice and judgment could also explain why pregnant individuals who are planning an elective CS without a medical indication rarely take part in antenatal classes:

“*The women who really want to have a caesarean section*,* i.e. for whom a vaginal birth is categorically ruled out*,* hardly ever attend a birth preparation course.*” Participant 4.

**Fig. 1:**
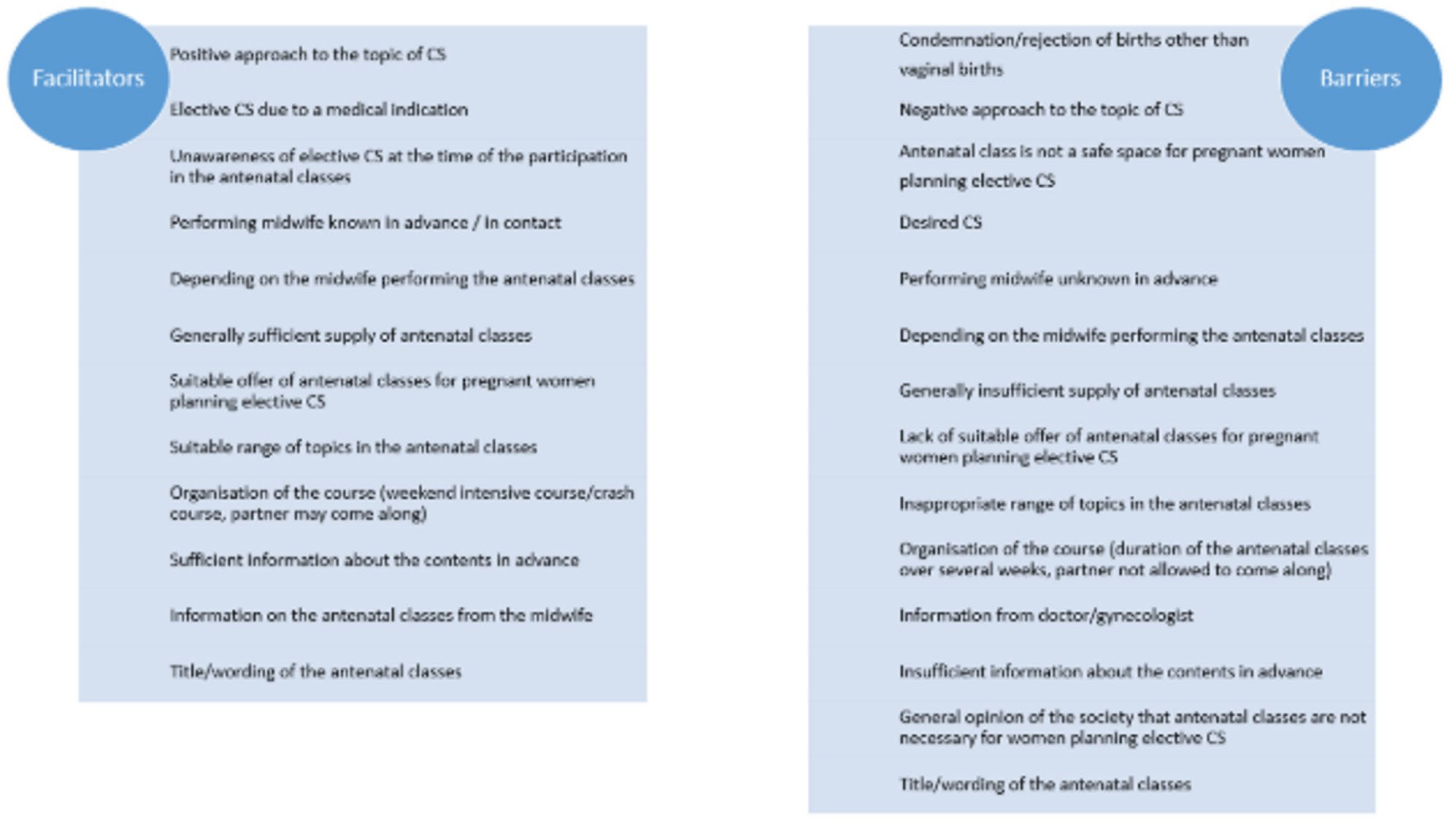
Barriers and facilitators for participation of pregnant individuals planning elective CS in antenatal classes

All the mentioned barriers and facilitators for attendance are listed in Fig. 1. In addition, the participants emphasized that regardless of the mode of birth, the pregnant individual’s level of education and financial situation influence participation in antenatal classes.

### Rationale for antenatal class attendance

Most midwives estimated that the majority of pregnant individuals in antenatal classes have clear birth preferences, mostly in favor of a vaginal birth. Nevertheless, many participants considered the participation of pregnant individuals preparing for an elective CS in antenatal classes to be relevant in principle. One of the reasons given for this is the very broad range of topics, which goes far beyond the mode of delivery. The midwives also pointed out that antenatal classes are important for the prenatal period. This is due to the information provided, such as nutrition and physical strengthening exercises as well as complaints during pregnancy. The midwives mentioned other reasons, such as the provision of information about CS, vaginal birth, networking with other parents-to-be, or information about the postnatal period. The following quote illustrates the need to attend an antenatal class regardless of the planned mode of birth:

“*I think that everyone should attend a birth preparation course because the course also covers other topics. In other words*,* it’s not just about the birthing process*,* but also about preparing for birth and parenthood. And then afterwards*,* the postnatal period*,* breastfeeding and so on. And there are still questions about the planned caesarean section. In other words*,* I would always highly recommend taking part*,* regardless of how you want to give birth.*” Participant 6.

Some midwives, conversely, argued that antenatal classes are primarily designed for pregnant individuals planning a vaginal birth. Further, it was stated that pregnant individuals preparing for an elective CS would benefit more from other preparations, and that they may feel uncomfortable with topics related to vaginal birth. The following quote illustrates why some women planning elective CS might feel uncomfortable in antenatal classes:

“*The need is there*,* but sometimes women who have already registered cancel because they realize that they have to have a planned caesarean section. They cancel for different reasons. One reason is because they say: ‘Well*,* some topics don’t interest me at all.’ Or let’s say: ‘They don’t affect me at all.’ Some also say: ‘Well*,* I think I might be sad or wistful when I hear how a ’normal birth’ works*,* what options there are. And I don’t even have the option of choosing this birth option.’ Disappointment or sadness is certainly also the reason why some people withdraw from a course.*” Participant 4.

This may reflect an implicit hierarchy of birth methods. In addition, the argument that other preparations might be more suitable suggests a potentially narrow conceptualization of antenatal classes, in which vaginal birth is seen as the standard. As a result, pregnant women planning an elective CS may not participate in this shared preparatory format and informational inequities may arise.

### Consideration of CS in general and elective CS in antenatal classes

All of the midwives stated that they generally take the topic of CS into account in their antenatal classes. The most common reason they gave for this is that CS can result from the course of labour and is therefore relevant for all pregnant individuals. Others cited the high CS rate, the participants’ desire for more information on CS or that the mode of birth is not yet known at the time of participation. Most of the midwives stated that they generally discuss the content of elective CS in the antenatal classes. One midwife pointed out that she would not include elective CS in her antenatal classes, because she thinks there is not much to address on the subject in advance:

“*I: Yes*,* so what content and aspects about the caesarean section are discussed?*

*P: The procedure*,* the anaesthetic*,* the wound healing afterwards*,* the changes in the puerperium. Everything to do with the caesarean section*,* how it is or how it results from the birth. There’s not much to say in advance about a planned caesarean section*,* because there’s not much that precedes it. It’s mainly the secondary caesarean section that we’re talking about*,* how it results from the birth. You can discuss the planned ones*,* but there’s not much to actually say.*

*I: Yes. What are the topics that are relevant for the planned section? So when you say there aren’t many?*

*P: Nothing really.*” Participant 9.

The assumption that there is not much to address shows that midwives’ assumptions play a role that might overlook emotional, psychological, and preparatory needs. Further, they assumed that the pregnant individual had already discussed the elective CS with the gynecologist, or they offered their own separate preparation for pregnant individuals preparing for an elective CS. This could indicate that some midwives see their role more in the discussion of vaginal birth, while the discussion of the elective CS is attributed more to the doctors. Others pointed out that they consider the topic as required, i.e. depending on enquiries or the number of pregnant individuals planning elective CS.

**Fig. 2:**
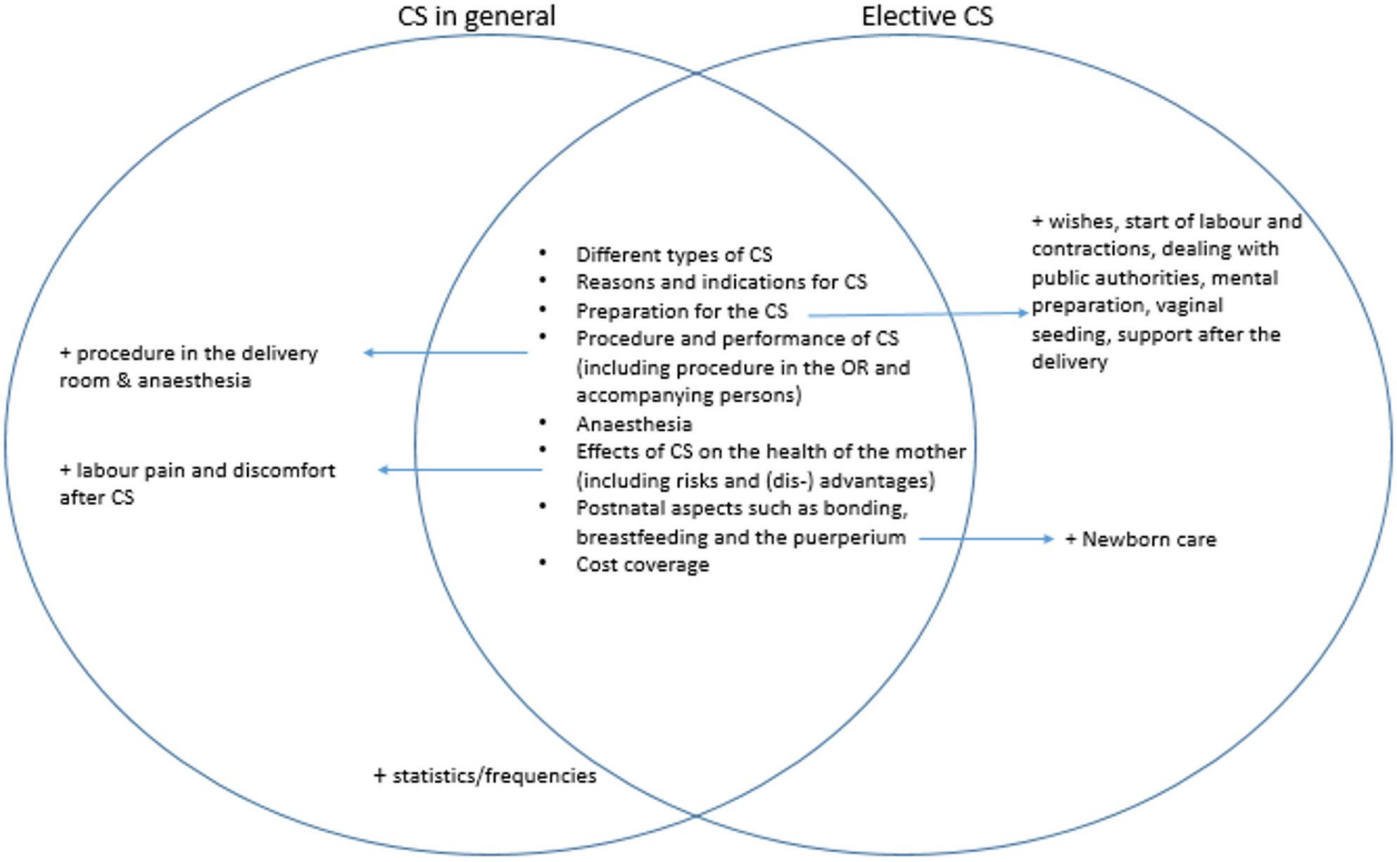
General consideration of topics relating to CS in general and elective CS

Figure 2 provides an overview on topics on CS in general and elective CS generally considered in the participants’ antenatal classes. Topics covered by midwives in antenatal classes in the context of both general CS and planned CS are shown at the intersection of the two circles. A ‘+’ outside the intersection indicates that the topic was only mentioned by the participating midwives in either the general or planned CS context. The figure exhibits a high degree of overlap in terms of which topics relating to CS in general and elective CS are generally addressed in the antenatal classes. With regard to elective CS, more topics on preparation are mentioned and the health effects relate only to the mother, not the newborn.

### Information needs of pregnant individuals

Some midwives reported that pregnant individuals preparing for an elective CS have more specific information needs on the topic of CS in general and elective CS in the antenatal classes than pregnant individuals planning a vaginal birth. Others, however, stated that the information needs of pregnant individuals preparing or not preparing for an elective CS do not differ, for example:

“*I think they are actually the same topics*,* I have to be honest. Because the need for counselling is actually exactly the same. Exactly. So*,* the women who are actually aiming for a normal birth tend to focus on why a caesarean section might be necessary despite a normal birth. And for women who already know that they need or want to have a caesarean section*,* the focus is more on these preventative and aftercare issues. Topics for the child*,* right? So*,* a little bit / The focus is a little bit different*,* where most of the questions come afterwards. But apart from that*,* the topics are actually the same.*” Participant 6.

Some midwives reported that pregnant individuals planning an elective CS ask few or no questions about CS in general as well as elective CS in the antenatal classes. The fact that pregnant individuals planning elective CS ask few or no questions in antenatal classes is not necessarily due to a lack of interest, but can be explained by a shift in the source of information on the subject of CS. This is in line with some participants explaining that these pregnant individuals obtain the information elsewhere, for example, in a one-to-one discussion or during the preoperative informed consent discussion, which is sufficient for some pregnant individuals. It is possible that pregnant individuals planning an elective CS would prefer to talk about elective CS in a protected space, possibly preferring to talk to doctors. For some, this could be a sensitive topic that they would rather not discuss in a group but in private. However, another possible explanation is that they do not find space for this in the antenatal classes. This fits in with the statement that pregnant individuals preparing for an elective CS often do not disclose their planned mode of birth in the antenatal classes, making it difficult to assess their information needs. Overall, the midwives pointed out that the topic of elective CS depends heavily on the respective antenatal classes and that there is correspondingly great heterogeneity with regard to the information provided:

“*I suppose that depends very much on the course. I know that there are courses where the caesarean section is almost not discussed at all or only as emergency management*,* which is not advisable. I think that leaves many*,* many questions or uncertainties*,* or perhaps even encourages uncertainties. I sometimes experience that women*,* I also work in a clinic*,* say after a caesarean section that they have heard in their antenatal classes that it is so bad for the child and / So*,* where I think: “Oh God!” So*,* I think it really depends on the course. And*,* well*,* I hope it’s different in my classes. But I don’t know that for sure*,* of course.*“ Participant 13.

### Modifications to address the information needs in the antenatal classes

The participants mentioned various modifications to improve addressing the information needs of pregnant individuals planning elective CS in antenatal classes. For example, they pointed out that the clinic often provides information about the operation at very short notice:

“*Well*,* the consultation about the caesarean section always takes place quite late. This means that it is usually only carried out shortly before the planned date of the caesarean section and the procedures are only discussed there. This means that the women otherwise only have an idea of how the whole thing will actually work at a very late stage*,* which of course also fuels fears.*” Participant 6.

Accordingly, one midwife suggested that a surgeon could be included in the course for the CS education section, although she doubted that this would be feasible in practice.

Nonetheless, some participants also spoke out against addressing the information needs of pregnant individuals preparing for an elective CS more strongly. They argued that CS should not be given even more attention, an increased focus could promote the uncertainties/fears of other pregnant individuals, and that antenatal classes already provide sufficient information about elective CS. For example, one midwife said:

“*And as I said*,* I personally don’t think that belongs in an antenatal class where everyone is sitting in*,* yes? That’s really something*,* I just have to say that it would only create unnecessary stress and anxiety and wouldn’t do any good*,* right?*” Participant 8.

The arguments point to deeper normative considerations. The comment that the CS should not receive too much attention points to a hierarchical understanding of birth modes. The argument that increased information about the planned CS could unsettle pregnant women highlights a group-oriented logic that may overcome the individual information needs of pregnant individuals planning an elective CS because they are in the minority.

**Fig. 3:**
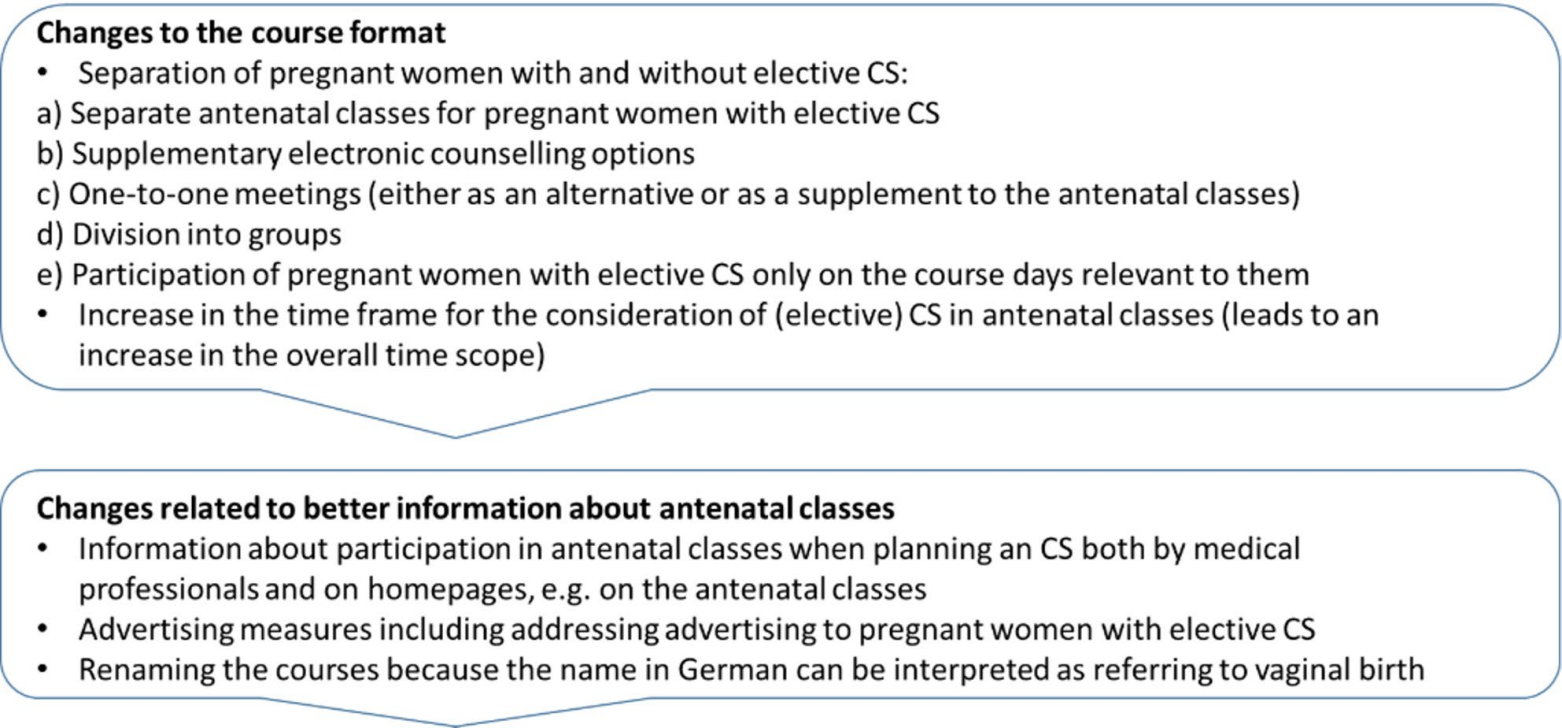
Changes to the course format to address the information needs

The midwives proposed several possibilities for change, most of which pertain to the course format in general or to improving the communication and visibility of antenatal classes, including both marketing and informational aspects. An overview of these can be found in Fig. 3.

The idea of offering a separate course was subject to extensive discussion during the interviews. The most frequently mentioned reason for offering separate antenatal classes for pregnant individuals preparing for an elective CS was that it would provide a safe space:

“*Because I could imagine that the safe space is a bit different*,* right? That they have the feeling that they are now perhaps among like-minded people or that they are all kind of in the same boat. So I can imagine that other topics easily come up as well. Maybe a bit more open discussion than / Because I said*,* no*,* up to now they’ve always been in the minority. If they talked about it at all*,* they were always in the minority. That’s why I could imagine that perhaps a course aimed at precisely that could be helpful.*” Participant 1.

One midwife indicated that she already offered separate antenatal classes for pregnant individuals preparing for an elective CS. Many of the midwives emphasized the uncertainty regarding the need and demand of pregnant individuals planning an elective CS for separate antenatal classes, especially in rural areas, and the possible lack of profitability for midwives. The midwives pointed out difficulties with planning, as the decision or medical indication for a CS is often made shortly before the expected date of birth. In view of this, they suggested that the antenatal classes should be offered either as shorter, multi-part evening courses or as an intensive weekend course at short notice before the planned date of birth.

## Discussion

The present study identified various barriers and facilitators for the participation of pregnant individuals preparing for an elective CS in antenatal classes. These include being uninformed about antenatal classes, how the midwife providing antenatal education deals with the topic of elective CS and class organization. Most participants also see a need for pregnant individuals planning an elective CS to participate in antenatal classes. The topic of CS is generally included in antenatal classes, and most participating midwives address the elective CS in their antenatal classes. The midwives mentioned several approaches to address the information needs of pregnant individuals preparing for an elective CS better. The idea of offering a separate course was subject to extensive discussion during the interviews. Some participants raised concerns regarding its practical feasibility.

Most midwives considered the participation of pregnant individuals preparing for an elective CS in antenatal classes as relevant. This is in line with other studies in which the participation of pregnant individuals in antenatal classes was also considered relevant for reasons other than the exclusive preparation for vaginal birth [2, 12]. It is emphasized that topics beyond vaginal birth in particular are of equal importance for pregnant individuals preparing for an elective CS as for all pregnant individuals, and that the networking aspect has the same importance to all pregnant individuals [3].

However, the midwives pointed out that not all potential participants are aware that many other topics beyond vaginal birth are covered in antenatal classes. Accordingly, some may mistakenly believe that it would not make sense for them to attend. These findings were also evident in a regional study of midwives, in which elective CS was given as a reason for non-participation in some cases [13]. We propose several approaches to counteract this possible lack of awareness.

The midwives considered it very important to signal an open approach to the topic of elective CS. This is likely to encourage pregnant individuals who are planning a CS to participate in antenatal classes. In the literature, a negative approach on the part of a midwife to a pregnant individual’s decision in favor of CS was observed [14]. In another study, vaginal birth was described as preferred and CS as less desirable, and that pregnant individuals felt stigmatized because of CS [15]. In the course of our interviews, there were repeated hints of a, possibly unconscious, hierarchical evaluation of the birth modes. Vaginal birth appears to be the ideal or natural mode of birth, while CS, and elective CS in particular, appears to be inferior. This is consistent with a qualitative evidence synthesis which found that in many cases vaginal birth is seen as ‘natural’ and that cultural aspects in many societies influence women to choose vaginal birth to avoid social sanctions [16]. The authors of a survey of Australian midwives and obstetricians about birth options and interventions during labor also concluded that clinicians need to be aware of their own beliefs and preferences regarding childbirth as a potential source of bias when counseling women [17]. In the course of a qualitative analysis, midwives were more critical of the topic of CS than gynecologists [18]. This also indicates that the hierarchical assessment differs depending on the professional group and this could be a reason why midwives occasionally referred to doctors with regard to the topic of elective CS.

All midwives indicated that they generally address the topic of CS in their antenatal classes. Similarly, Murphy et al. recommended informing about the possibility of a CS and its associated implications, even when a vaginal birth is planned [19]. In line with this, we were able to show that the midwives also address some aspects of elective CS in their antenatal classes.

Study participants repeatedly emphasized that certain content can be overwhelming or inappropriate for different groups of pregnant individuals and therefore lead to individuals feeling anxiety or discomfort. This was mentioned both in the context of pregnant individuals who are planning an elective CS and receive information on vaginal birth, as well as pregnant individuals who are planning a vaginal birth and receive information on a CS. This reveals an area of tension between the individual needs of the respective groups. As the majority of pregnant individuals are planning a vaginal birth, their needs are likely to be prioritized over the needs of pregnant individuals planning an elective CS when it comes to a trade-off. However, studies have shown that reliable information on complications during labour can reduce anxiety [12]. Therefore, the quality of the information seems to be important here, indicating that missing or inadequate information can increase anxiety [20]. Given that the midwives reported barriers to attending antenatal classes and noted that their specific information needs were not always adequately addressed, it seems important to ensure that pregnant individuals preparing for an elective CS also have access to tailored, high-quality information. The authors of a review found that many pregnant women search for health-related information online. While they noted various advantages, such as quick and easy access, they also highlighted concerns regarding the reliability of the information [21].

A necessary step in this direction is increasing the overall number of antenatal classes in general, regardless of the planned mode of birth. This is consistent with the finding that the insufficient availability of antenatal classes was cited as a barrier to participation and would benefit all pregnant individuals and provide access to high-quality information. In a regional midwife study conducted in 2018, around 44% of the midwives surveyed stated that they had a significant excess demand for antenatal classes [22]. In another regional midwife study from 2015, 75% of the midwives stated that there was excess demand [13].

One suggestion about greater considerations of the information needs of pregnant individuals planning an elective CS involves separating pregnant individuals preparing for an elective CS from other pregnant individuals, sometimes only for individual units of the course, but sometimes also by offering a separate course only for pregnant individuals planning an elective CS. Some doubted that there was sufficient demand and that separate courses could be realized economically. Municipalities could support midwives in organizing antenatal classes by providing free rooms in order to achieve economic efficiency even with a low number of participants. This has already been realized in individual municipalities in the past [23]. Another option is offering digital antenatal classes. Even though the participating midwives only mentioned supplementary digital counseling options and did not suggest a completely digital course, this can be an approach to offering such courses economically, especially in more rural areas [24]. Conversely, there are consistent reports across various cultures that there is a preference for face-to-face, non-electronic education [25]. Overall, the existence of separate antenatal classes for pregnant individuals preparing for an elective CS (one participant already offers such courses) shows that the implementation of such antenatal classes is possible in principle. On the one hand, this proposal could further promote the separation between pregnant individuals planning an elective CS and pregnant individuals planning a vaginal birth, thus increasing possible stigmatization and reducing exchange between the two groups. Conversely, the fact that antenatal classes are not a safe space for pregnant individuals planning an elective CS and that birth modes other than vaginal birth are partially rejected was cited as a barrier to participation. In the course of the interviews, there were indications that pregnant individuals obtain information about elective CS in ways other than antenatal classes and that some individuals do not disclose their mode of birth. A separate course could counteract such mechanisms. Nonetheless, it would be very important to hear the perspective of affected pregnant individual’s and to evaluate whether such courses would be successful in increasing the participation of pregnant individuals planning an elective CS and whether they have a positive effect on their level of knowledge.

### Strengths and limitations

In this study, we followed a protocol that had been established and published in advance, ensuring that our approach was transparent and rigorous. We were thus able to gain valuable insights into how antenatal classes address elective CS and to identify factors influencing the participation of pregnant individuals preparing for an elective CS.

However, it is important to emphasize that midwives could only indicate which barriers or facilitators they think pregnant individuals experience. Further, we assume that a considerable number of pregnant individuals preparing for an elective CS may not participate in antenatal classes. Therefore, the information needs of pregnant individuals presented here can be incomplete and distorted by the subjective interpretation of the midwives. To round out the picture, we are also conducting interviews with pregnant individuals who are preparing for an elective CS; the protocol has already been published [26].

Since the study is part of a master’s thesis, the interviewer had no prior experience in conducting qualitative interviews. However, a person with expertise in qualitative methods provided supervision.

Overall, the study comprises a small sample, which is common in qualitative research methods. A systematic review suggests that data saturation can be reached even with small sample sizes (9–17 interviews) [27]. In the course of the interviews it emerged that no further topics were added, meaning that we reached data sufficiency.

Presumably, the majority of midwives who took part in the study had already dealt with the consideration of elective CS in antenatal classes in advance, were interested in the topic or were open to it. In addition, we carried out convenience sampling. Accordingly, it may be that, for example, a greater need was seen to align antenatal classes with the needs of pregnant individuals planning elective CS. Nevertheless, we could include a sample with a high degree of heterogeneity, for example in terms of age and professional experience. We could not recruit a male midwife, but this can also be explained by the gender-specific distribution in this female-dominated occupational group [28, 29]. The heterogeneity indicates that we could include midwives with different perspectives and opinions. In addition, due to the fact that the study was conducted in the context of a master’s thesis with limited capacity, we limited ourselves to including midwives who already had experience with pregnant individuals planning an elective CS in their antenatal classes. However, to complete the picture, the perspective of midwives who have no such experience would also be relevant.

Since the midwives knowingly took part in interviews, we cannot rule out that the response behavior was (unknowingly) influenced at some points. However, the analysis shows that it was possible to include critical views.

We did not base the interview guide on a formal theoretical framework. However, we performed a pretest with a midwife to ensure comprehensibility and completeness.

### Implications for research and practice

Currently, we pursue the supplementary conduct of interview studies with pregnant individuals planning an elective CS. The aim is to gain a comprehensive perspective on their informational needs and prior experiences within the context of antenatal classes. If the interviews confirm the need for a stronger focus on addressing their informational requirements, as several participants in the current study suggested, the modifications proposed in this paper should be implemented and evaluated. This paper suggests which topics should be addressed in detail. For example, as part of a research project, a separate course for pregnant individuals planning a CS could be developed. The development of this course should involve pregnant individuals, midwives, and obstetrics to ensure alignment with the needs of the target group. Additionally, it would allow evaluation of ways to design such an offering in a feasible manner. A subsequent evaluation could assess its practical feasibility.

## Conclusion

The present study with midwives identifies various barriers and facilitators for the participation of pregnant individuals preparing for an elective CS in antenatal classes. These include lacking information about the content covered in antenatal classes, how the midwife providing the antenatal education classes deals with the topic of elective CS and the implementation of antenatal classes. In summary, a more comprehensive education regarding the content of antenatal classes is necessary. Most participants also see a need for pregnant individuals planning an elective CS to participate in antenatal classes. The topic of CS is generally included in the antenatal classes, and most of the participating midwives address the elective CS in their antenatal classes. They mentioned several approaches to better address the information needs of pregnant individuals preparing for an elective CS. The idea of offering a separate course was subject to extensive discussion during the interviews. Some participants raised concerns regarding its practical feasibility. Following additional research on the perspective of pregnant individuals, potential modifications to enhance targeted support, such as the implementation of a separate course, should be evaluated.

## Supplementary Information

Supplementary Material 1.

Supplementary Material 2.

Supplementary Material 3.

## Data Availability

The datasets used and/or analyzed during the current study are available from the corresponding author on reasonable request.

## Abbreviations

CS: caesarean section
IFOM: Institute for Research in Operative Medicine
