# Supplementary material for: Midwives’ perspective on participation of pregnant individuals planning an elective caesarean section delivery in antenatal classes in Germany: a qualitative interview study
Source: BMC Pregnancy Childbirth. 2026 Jul 21;26:791. doi: 10.1186/s12884-026-09676-z (PMC13386846; doi:10.1186/s12884-026-09676-z)
Supplement: Supplementary file 1 — Supplementary Material 1. [file 12884_2026_9676_MOESM1_ESM.docx]

**Supplementary material 3. Coding tree**

| **Content** | **Main category** | **Category** | **Subcategory** | **Rule** | **Specification** |
| --- | --- | --- | --- | --- | --- |
| **General information about the antenatal classes (black)** | Type of antenatal class | General antenatal class according to §134 SGB V |  | Single word | Type of antenatal class offered by the midwife interviewed |
|  |  | Other type of antenatal class |  | Single word |  |
|  | Scope of the antenatal class | Temporal |  | In hours | Time frame and group size of the antenatal class |
|  |  | Group size |  | Number |  |
|  | Participants in antenatal classes | Preference of the pregnant woman regarding the type of delivery | Preference  yes/no | Number/ percentage | Proportion of pregnant individuals who have already decided or not decided on their type of delivery at the start of the antenatal class |
|  |  |  | Vaginal vs. Caesarean section | Number/ percentage | Distribution of pregnant individual's preferences between caesarean sections and vaginal births |
|  |  | Average number of pregnant individuals planning elective caesarean sections |  | Number | Average number of pregnant individuals in antenatal classes planning elective caesarean sections |
| **The topic of caesarean sections in general and elective caesarean sections in antenatal classes (blue)** | Consideration of the topic of caesarean sections in antenatal classes | General consideration of the topic of caesarean sections |  | Single word | General consideration for the topic of caesarean sections in antenatal classes |
|  |  | Consideration of the topic of caesarean sections only if required by the participants |  | Context | Aspects/contents of the caesarean section that are not fundamentally taught in the antenatal class |
|  |  | Emphasised relevant content on caesarean section |  | Context | Relevant content that the midwife emphasises on the topic of caesarean section in the antenatal class |
|  | Consideration of the topic of elective caesarean sections in antenatal classes | General consideration of the topic of elective caesarean sections |  | Single word | General consideration of the topic of elective caesarean sections in antenatal classes |
|  |  | Consideration of the topic of elective caesarean sections only if required by the participants |  | Context | Aspects/content relating to elective caesarean sections that are not genereally taught in the antenatal class |
|  |  | Relevant content on elective caesarean sections |  | Context | Contents that the midwife finds relevant on the topic of elective caesarean section in the antenatal class |
|  | Use of resources/materials to provide information |  |  | Context | Resources and materials used to inform about the caesarean sections in general and elective caesarean sections |
|  | Proportion of time spent on the topic of caesarean sections in general and elective caesarean sections | Proportion of time spent on the topic caesarean section |  | In hours/ minutes | Time spent on the topic of caesarean sections in antenatal classes |
|  |  | Proportion of time spent on the topic of elective caesarean sections |  | In hours/ minutes | Time spent on the topic of elective caesarean sections in antenatal classes |
| **Rationale for antenatal class attendance of pregnant individuals planning an elective caesarean section (green)** | Assessment of the need for pregnant individuals planning elective caesarean sections to participate |  |  | Context | Midwife's assessment of the need for pregnant individuals planning elective caesarean sections to attend antenatal classes |
|  | Reasons for the decision of pregnant individuals planning elective caesarean sections to participate |  |  | Context | Reasons according to the midwife that speak in favour of pregnant individuals with an elective caesarean section taking part in antenatal classes |
|  | Reasons for the decision against participation of pregnant individuals planning elective caesarean sections |  |  | Context | Reasons according to the midwife that speak against the participation of pregnant individuals with an elective caesarean section in antenatal classes |
|  | Fulfilment of expectations of pregnant individuals planning elective caesarean sections |  |  | Context | Midwife's assessment of the extent to which the expectations of pregnant individuals planning elective caesarean sections were met in antenatal classes |
| **Information needs of pregnant individuals in antenatal classes on caesarean sections in general and elective caesarean sections (yellow)** | Interesting topics for pregnant individuals on caesarean sections | Frequently asked questions/aspects |  | Context | Questions and aspects from pregnant individuals that arise particularly frequent in relation to caesarean sections in antenatal classes |
|  |  | Frequently mentioned insecurities/fears |  | Context | Insecurites/fears on the part of the pregnant individuals, which are mentioned particularly frequent in the antenatal classes in relation to the caesarean section |
|  | Questions/topics raised by pregnant individuals with (consideration of) elective caesarean section | Frequently asked questions |  | Context | Questions about the caesarean section that pregnant individuals planning elective caesarean sections ask particularly frequent; including questions that go beyond the surgical procedure |
|  |  | Frequently mentioned insecurities/fears |  | Context | Insecurities or fears on the part of pregnant individuals planning an elective caesarean section, which are mentioned particularly frequent in relation to the planned caesarean section |
|  | Unanswered questions/topics from pregnant individuals planning elective caesarean sections |  |  | Context | Questions and/or aspects that remain unanswered/unconsidered in antenatal classes for pregnant individuals planning elective caesarean sections, according to the midwife |
| **Addressing the information needs of pregnant individuals planning elective caesarean sections in antenatal classes (red)** | Approaches to increase addressing the information needs of pregnant individuals planning elective caesarean sections | Own concrete ideas/proposals | Own proposals for changes to the class format | Context | Midwife’s own concrete suggestions/ideas for strengthening the information needs of pregnant individuals planning elective caesarean sections by changing the class format |
|  |  |  | Own proposals for changes to the class content |  |  |
|  |  |  | Other proposals for changes | Context | Midwife’s own concrete suggestions/ideas for strengthening the information needs of pregnant individuals planning elective caesarean sections by changing the class content |
|  |  |  | Realistic proportion of time required to implement the above proposals | Context | Other specific suggestions/ideas of the midwife to strengthen the information needs of pregnant individuals planning elective caesarean sections in antenatal classes |
|  |  |  | Aspects required to implement the mentioned proposals | Context | Other specific suggestions/ideas of the midwife to enhance the information needs of pregnant individuals planning elective caesarean sections in antenatal classes |
|  |  |  |  | Context | Midwife's assessment of what is necessary for the implementation of their suggestions |
|  |  | Opinion on the proposed modular class format |  | Context | Midwife's opinion on proposed modular class format, e.g. in the form of classes specifically designed to meet the information needs of pregnant individuals who have opted for a planned caesarean section |
|  |  | Implementation of the proposed modular class format in practice |  | Context | Midwife's assessment of the implementation of a modular class format in practice, for example in the form of hours specifically geared to the information needs of pregnant individuals who have opted for a planned caesarean section |
|  | Aspects/topics to be prioritised for elective caesarean section |  |  | Context | Topics/aspects of the elective caesarean section that should be prioritised in the antenatal class according to the midwife |
|  | No ideas/proposals for an improved addressing of the information needs of pregnant individuals planning elective caesarean sections |  |  | Context | Midwife has no other specific suggestions/ideas to strengthen the informational needs of pregnant individuals planning an elective cesarean section in the antenatal class |
| **Factors influencing the participation of pregnant individuals planning elective caesarean sections in antenatal classes (pink)** | Factors hindering the participation of pregnant individuals planning elective caesarean sections |  |  | Context | Midwives' perceptions of factors that negatively influence the participation of pregnant individuals planning elective caesarean sections in antenatal classes |
|  | Promoting factors for the participation of pregnant individuals planning elective caesarean sections |  |  | Context | Midwife's perception of factors that positively influence the participation of pregnant individuals planning elective caesarean sections in antenatal classes |
| **Additions to the consideration of information needs of pregnant individuals planning elective caesarean sections in antenatal classes (violet)** | Aspects that have not yet been considered but are perceived as important |  |  | Context | Additions to the topic from the midwife's side that have not been taken into account so far but are considered important by the midwife |
