# Supplementary material for: Midwives’ perspective on participation of pregnant individuals planning an elective caesarean section delivery in antenatal classes in Germany: a qualitative interview study
Source: BMC Pregnancy Childbirth. 2026 Jul 21;26:791. doi: 10.1186/s12884-026-09676-z (PMC13386846; doi:10.1186/s12884-026-09676-z)
Supplement: Supplementary file 2 — Supplementary Material 2. [file 12884_2026_9676_MOESM2_ESM.docx]

**Supplementary material 1. Interview guide**

Preparation and introduction

Hello Mrs/Mr (name),

Thank you for taking the time to conduct this interview with me today.

As I have already explained to you in advance, I would like to assess the information needs of pregnant women with a planned (elective) caesarean section and how these can be addressed in antenatal classes. I am interested in your assessment as an expert of this. Please relate your answer in the interview to pregnant women with an elective caesarean section and pregnant women who are planning a caesarean section due to a medical indication. Do you have any questions about this in advance, or was the information provided so far clear to you?

(If the answer is no or if all questions have been clarified)

Very good. Before we start the interview, I would like to clarify a few organisational aspects with you.

Emphasis of the voluntary nature of participation in the interview: The questions in the interview are all voluntary. If you do not wish to answer a question, you can do so without giving a reason. You can also cancel the interview at any time without giving a reason. There will be no disadvantages for you at any time. Please let me know if this is your wish. There are no right or wrong answers. Your experiences and opinions on the subject are of relevance.

Confidentiality and data protection: You have already signed the consent and data protection declaration in advance and know that the interview will be recorded, so that the data can be written down and analysed afterwards. Your data will be treated confidentially and used exclusively for this study. Your data will be anonymised as part of the study so that no conclusions can be drawn about you.

Do you have any further questions? I would like to make sure again: Do you consent to the interview being recorded?

(In case of consent)

Great, if there are no more questions, we can start the interview. I'll switch on the recorder briefly. (**Starts recording device**)

**General information on the antenatal classes**

(First, I would like to talk about a few general aspects of antenatal classes.)

| **Nr.** | **Main question** | **Detailed question** |
| --- | --- | --- |
| 1 | General information about antenatal classes | - Do you offer antenatal classes that are billable according to §134 SGB V? (health insurance service according to the midwife contract) Do you also offer other (private, specific) antenatal classes? - What is the scope of your antenatal classes? (Group size, time - according to midwife contract: maximum 14 hours, maximum 10 pregnant women) - How many of the pregnant women already have a preference regarding the type of delivery at the beginning of the antenatal classes? What is the approximate distribution between vaginal births and caesarean sections? - How many pregnant women with a planned caesarean section occur in your courses on average? |

**The (planned) caesarean section in the antenatal classes**

| **Nr.** | **Main question** | **Detailed question** |
| --- | --- | --- |
| 2 | To what extent is the caesarean section taken into account in your antenatal classes?  Feel free to refer your answers to both caesarean sections in general and planned caesarean sections. | - Is a caesarean section always discussed in the antenatal course or only if the participants require it? How is this with regards to the planned caesarean section? - Which content and aspects do you emphasize in your birth preparation courses that are particularly relevant for caesarean births? What content and aspects are particularly relevant for planned caesarean births? - Which resources or materials do you use to provide information about (planned) caesarean sections? - How much time do you estimate the topic of caesarean sections to take up in (14) hours? And to which extent is time spent on elective caesarean sections? |

**Demand for participation in antenatal classes for pregnant women with a planned caesarean section**

| **Nr.** | **Main question** | **Detailed question** |
| --- | --- | --- |
| 3 | How do you assess the need for pregnant women with a planned caesarean section to attend antenatal classes? | - For what reasons would you say that pregnant women with a planned caesarean section are more likely to decide in favour of or against attending an antenatal course? - Withouth experience: based on conversations with colleagues, how many pregnant women with a planned caesarean section attend antenatal courses? - With experience: To what extent do you feel that the expectations of pregnant women with a planned caesarean section have been fulfilled in the antenatal courses? |

**Information needs of pregnant women with a planned caesarean section in antenatal classes**

| **Nr.** | **Main question** | **Detailed question** |
| --- | --- | --- |
| 4 | I general, what topics relating to caesarean sections are of interest to pregnant women in antenatal classes? | - Are there certain questions from pregnant women in your antenatal courses that arise frequently, in particular to the caesarean section? - Are there specific insecurities or fears of pregnant women that are mentioned frequent in the antenatal course, relating to the caesarean section? |
| 5a | In case of experience: In contrast to this: What topics and questions do pregnant women who are planning or considering a caesarean section bring up in antenatal classes? | - Are there certain questions regarding the caesarean section that pregnant women with a planned caesarean section ask you particularly often? - Are there certain insecurities or fears of pregnant women that are mentioned frequently in relation to the planned caesarean section? - (If only the surgical procedure was discussed in advance): Which questions that go beyond the surgical procedure are frequently asked by pregnant women with a planned caesarean section in antenatal classes? - Which questions or topics do you think remain unanswered for pregnant women with a planned caesarean section in antenatal classes? |
| 5b | Without experience: What topics and questions do you consider relevant for pregnant women planning or considering a caesarean section in antenatal classes? | Which questions or topics do you think remain unanswered for pregnant women with a planned caesarean section in antenatal classes? |

**Addressing information needs in antenatal classes**

| **Nr.** | **Main question** | **Detailed question** |
| --- | --- | --- |
| 6 | In your opinion, how could the information needs of pregnant women with a planned caesarean section be addressed better in antenatal classes? | - Do you have specific suggestions and ideas? - Are there any specific adjustments or changes to the course content or format that could be useful for pregnant women with a planned caesarean section? - Realistically, how much time would be needed to implement your proposals? - In your opinion, what would be necessary to implement the proposals or ideas? |
| 7 | In your opinion, which topics or aspects of the planned caesarean section should be prioritised in the antenatal course? | - Why do you consider these to be relevant in particular? |
| 8 | What do you think of the idea of designing modular course formats? For instance as classes that are specifically designed to meet the information needs of pregnant women who have decided to have a planned caesarean section? | - Do you think that would be possible to implement into common practice? |

**Factors influencing the participation of pregnant women with a planned caesarean section in antenatal classes**

| **Nr.** | **Main question** | **Detailed question** |
| --- | --- | --- |
| 9 | In your opinion, what factors influence the participation or non-participation of pregnant women with a planned caesarean section in antenatal classes? | - Which factors do you consider to be favourable? - Which factors do you consider to be hindering? |
