# Supplementary material for: Midwives’ perspective on participation of pregnant individuals planning an elective caesarean section delivery in antenatal classes in Germany: a qualitative interview study
Source: BMC Pregnancy Childbirth. 2026 Jul 21;26:791. doi: 10.1186/s12884-026-09676-z (PMC13386846; doi:10.1186/s12884-026-09676-z)
Supplement: Supplementary file 3 — Supplementary Material 3. [file 12884_2026_9676_MOESM3_ESM.docx]

Supplementary material 2: COREQ checklist

**Consolidated criteria for reporting qualitative studies (COREQ) [1]**

Table 1: COREQ: 32-item checklist

| **Domain 1: Research team and reflexivity** | |
| --- | --- |
| *Personal characteristics* | |
| 1. Interviewer/facilitator  Which author/s conducted the interview or focus group? | 2.6 Data collection  “SJ, who was a Master's student in Prevention, Sports Therapy and Health Management and an employee at IFOM at the time the interviews were carried out, conducted the semi-structured interviews as part of her Master's thesis. At the time of the interviews, she had no prior formal training in conducting qualitative studies and was supervised by an individual with experience in conducting qualitative studies (NK).” |
| 2. Credentials:  What were the researcher’s credentials? E.g. PhD, MD |  |
| 3. Occupation:  What was their occupation at the time of the study? |  |
| 4. Gender:  Was the researcher male or female? |  |
| 5. Experience and training:  What experience or training did the researcher have? |  |
| *Relationship with the participants* | |
| 6. Relationship established:  Was a relationship established prior to study commencement? | 2.6 Data collection  “There was no relationship between the participants and the interviewer. The participants only knew that she was a Master's student and employee at IFOM and was conducting the study as part of her master's thesis.” |
| 7. Participant knowledge of the interviewer: What did the participants know about the researcher? e.g. personal goals, reasons for doing the research | 2.6 Data collection  *“*The participants only knew that she was a Master's student and employee at IFOM and was conducting the study as part of her master's thesis.” |
| 8. Interviewer characteristics:  What characteristics were reported about the interviewer/facilitator? e.g. Bias, assumptions, reasons and interests in the research topic | 2.6 Data collection  “SJ, who was a Master's student in Prevention, Sports Therapy and Health Management and an employee at IFOM at the time the interviews were carried out, conducted the semi-structured interviews as part of her Master's thesis.” |
| **Domain 2: study design** | |
| *Theoretical framework* | |
| 9. Methodological orientation and Theory: What methodological orientation was stated to underpin the study? e.g. grounded theory, discourse analysis, ethnography, phenomenology, content analysis | 2.7 Data analysis  We based the interview analysis on Mayring’s qualitative content analysis [10] using MAXQDA 2022 (VERBI Software, Berlin, Germany) [11]. |
| *Participant selection* | |
| 10. Sampling:  How were participants selected? e.g. purposive, convenience, consecutive, snowball | 2.4 Sample and sampling  “We included a convenience sample of midwives who have experience in conducting antenatal classes in Germany and who offered antenatal classes at the time of recruitment. Only midwives who had experience with pregnant individuals preparing for an elective CS attending at least one of their antenatal classes were included. It was possible to recruit enough midwives who met these criteria. Elective CS included both the maternal request CS and the elective CS due to a medical indication. If it had not been possible to recruit enough midwives with experience, we would have recruited midwives with no experience of pregnant individuals preparing for an elective CS in their antenatal classes. Sample size was determined by data sufficiency, with recruitment continuing until data were deemed adequate to comprehensively address the research questions. We used convenience sampling and data sufficiency because the study was part of a Master's thesis, which meant that we had limited time and resources.  To recruit midwives, midwives practicing in Germany were contacted by phone or email, or via invitation from midwifery associations. Recruitment took place from October to December 2023. We obtained the contact details of the midwives from the midwives' list of the GKV-Spitzenverband (National Association of Statutory Health Insurance Funds) [9]. Influencers in the field of midwifery science were contacted via Instagram and invited to share information about the study. This included both midwives who are active on social media and organizations. In addition, we leveraged the Institute for Research in Operative Medicine (IFOM) network to support recruitment and disseminated information about the study via online messaging platforms and social media accounts. There were no financial incentives for participation.  Midwives who were interested in participating received an email with the following study documents, prior to the interview: participation information, consent form and privacy statement. Furthermore, the email requested information on the characteristics relevant to heterogeneous composition of the sample, such as age, gender, and professional experience in conducting antenatal classes.” |
| 11. Method of approach:  How were participants approached? e.g. face-to-face, telephone, mail, email Reasons? | 2.6 Data collection  “We used an audio recording device to record the telephone interviews, which were then transcribed by an external service provider called Abtipper.de.” |
| 12. Sample size:  How many participants were in the study? | 2.4 Sample and sampling  “However, data sufficiency occurred after 15 interviews had been conducted. Subsequently, we conducted no further interviews. The interviews had a mean length of 27 minutes (range 14-52).” |
| 13. Non-participation:  How many people refused to participate or dropped out? Reasons? | 2.4 Sample and sampling  “Seven of the 31 interested midwives could not be included because they lacked experience in providing antenatal classes or working with pregnant individuals preparing for an elective CS in their antenatal classes (n=2), lacked time capacities (n=4), or were uncomfortable with the way the study was conducted (participant did not want any interview/audio recordings; n=1). Nine of the interested midwives did not further respond. However, data sufficiency occurred after 15 interviews had been conducted. Subsequently, we conducted no further interviews.” |
| Setting |  |
| 14. Setting of data collection:  Where was the data collected? e.g. home, clinic, workplace | 2.6 Data collection  “We conducted the pretest and the interviews by phone with the interviewer in a private environment at home or work, there was no guarantee that participants were necessarily alone at the time of the interview.” |
| 15. Presence of non-participants:  Was anyone else present besides the participants and researchers? |  |
| 16. Description of sample:  What are the important characteristics of the sample? e.g. demographic data, date | 3.1 Participants’ characteristics  “All participants were female and the mean age was 39 years (range 23-60). They had an average work experience of 11 years (range 1-25).” |
| *Data collection* | |
| 17. Interview guide:  Were questions, prompts, guides provided by the authors? Was it pilot tested? | 2.5 Development of the interview guide  “The interview guide consisted of 6 sections: 1) General information about the antenatal classes, 2) the topic of CS in general and elective CS in the antenatal classes, 3) rationale for antenatal class attendance for those planning elective CS, 4) information needs of pregnant individuals preparing for an elective CS in antenatal classes, 5) addressing the information needs in antenatal classes, 6) factors influencing the participation of pregnant individuals preparing for elective CS in antenatal classes. The interviews were conducted in German, the translated interview guide can be found in supplementary material 1.” |
| 18. Repeat interviews:  Were repeat interviews carried out? If yes, how many? | 2.6 Data collection  “and we did not repeat any interviews.” |
| 19. Audio/visual recording:  Did the research use audio or visual recording to collect the data? | 2.6 Data collection  “We used an audio recording device to record the telephone interviews, which were then transcribed by an external service provider called Abtipper.de.” |
| 20. Field notes:  Were field notes made during and/or after the interview or focus group? | 2.6 Data collection  “Reflexivity was addressed through repeated transcript review, during which the interviewer ensured accuracy of the transcripts, familiarized with the data, and critically examined potential biases and assumptions that may have influenced data collection and interpretation. In addition, parts of the analysis were conducted by two people, thereby minimizing personal biases and assumptions. Systematic documentation of reflexive considerations in the form of field notes or a reflexivity journal was not conducted.” |
| 21. Duration:  What was the duration of the interviews or focus group? | 2.4 Sample and sampling  “The interviews had a mean length of 27 minutes (range 14-52).” |
| 22. Data saturation:  Was data saturation discussed? | 2.4 Sample and sampling  “Sample size was determined by data sufficiency, with recruitment continuing until data were deemed adequate to comprehensively address the research questions. We used convenience sampling and data sufficiency because the study was part of a Master's thesis, which meant that we had limited time and resources.  […]  . However, data sufficiency occurred after 15 interviews had been conducted. Subsequently, we conducted no further interviews.” |
| 23. Transcripts returned:  Were transcripts returned to participants for comment and/or correction? | 2.6 Data collection  “The participants were not given the opportunity to correct or comment on the transcripts, and we did not repeat any interviews.” |
| **Domain 3: analysis and findings** | |
| *Data analysis* | |
| 24. Number of data coders:  How many data coders coded the data? | 2.7 Data analysis  “SJ and NK independently coded three interviews using the main categories. They discussed deviations to reach consensus. The interviewer then continued the first step (main categories) of coding independently. Both independently conducted the subsequent inductive coding based on 3 interviews. Following this, SJ and NK discussed the results to reach a consensus. During this step, the deductive category system from the first step was inductively refined and supplemented by further categories and subcategories. Then, the interviewer conducted the more in-depth coding independently.” |
| 25. Description of the coding tree:  Did authors provide a description of the coding tree? | The coding system can be found in supplementary material 3. |
| 26. Derivation of themes:  Were themes identified in advance or derived from the data? | 2.7 Data analysis  “We based the interview analysis on Mayring’s qualitative content analysis [10] using MAXQDA 2022 (VERBI Software, Berlin, Germany) [11]. The aim is to structure the transcribed interviews into main and sub-categories in order to analyze the material in a controlled and step-by-step manner. The development of the main and sub-categories is based on both a deductive and an inductive approach. In the deductive approach, main categories are defined based on the interview guide. In the inductive approach, additional categories are derived from the data material.” |
| 27. Software:  What software, if applicable, was used to manage the data? | 2.7 Data analysis  “We based the interview analysis on Mayring’s qualitative content analysis [10] using MAXQDA 2022 (VERBI Software, Berlin, Germany) [11].” |
| 28. Participant checking:  Did participants provide feedback on the findings? | 2.6 Data collection  “The participants were not given the opportunity to correct or comment on the transcripts, and we did not repeat any interviews.” |
| *Reporting* | |
| 29. Quotations presented:  Were participant quotations presented to illustrate the themes / findings? Was each quotation identified? e.g. participant number | Several quotations are presented within the results section. |
| 30. Data and findings consistent:  Was there consistency between the data presented and the findings? | Major themes are highlighted by the layout in the results section and numbering. |
| 31. Clarity of major themes:  Were major themes clearly presented in the findings? | See headings of the results section |
| 32. Clarity of minor themes:  Is there a description of diverse cases or discussion of minor themes? | Example: “Some midwives, conversely, argued that antenatal classes are primarily designed for pregnant individuals planning a vaginal birth. Further, it was stated that pregnant individuals preparing for an elective CS would benefit more from other preparations, and that they may feel uncomfortable with topics related to vaginal birth.” |

1. Tong A, Sainsbury P, Craig J. Consolidated criteria for reporting qualitative research (COREQ): a 32-item checklist for interviews and focus groups. Int J Qual Health Care. 2007;19(6):349-57. Epub 20070914. doi: 10.1093/intqhc/mzm042. PubMed PMID: 17872937.
